# Supplementary material for: Enhancing regeneration after acute kidney injury by promoting cellular dedifferentiation in zebrafish
Source: Dis Model Mech. 2019 Apr 5;12(4):dmm037390. doi: 10.1242/dmm.037390 (PMC6505474; doi:10.1242/dmm.037390)
Supplement: Supplementary information [file dmm-12-037390-s1.pdf]

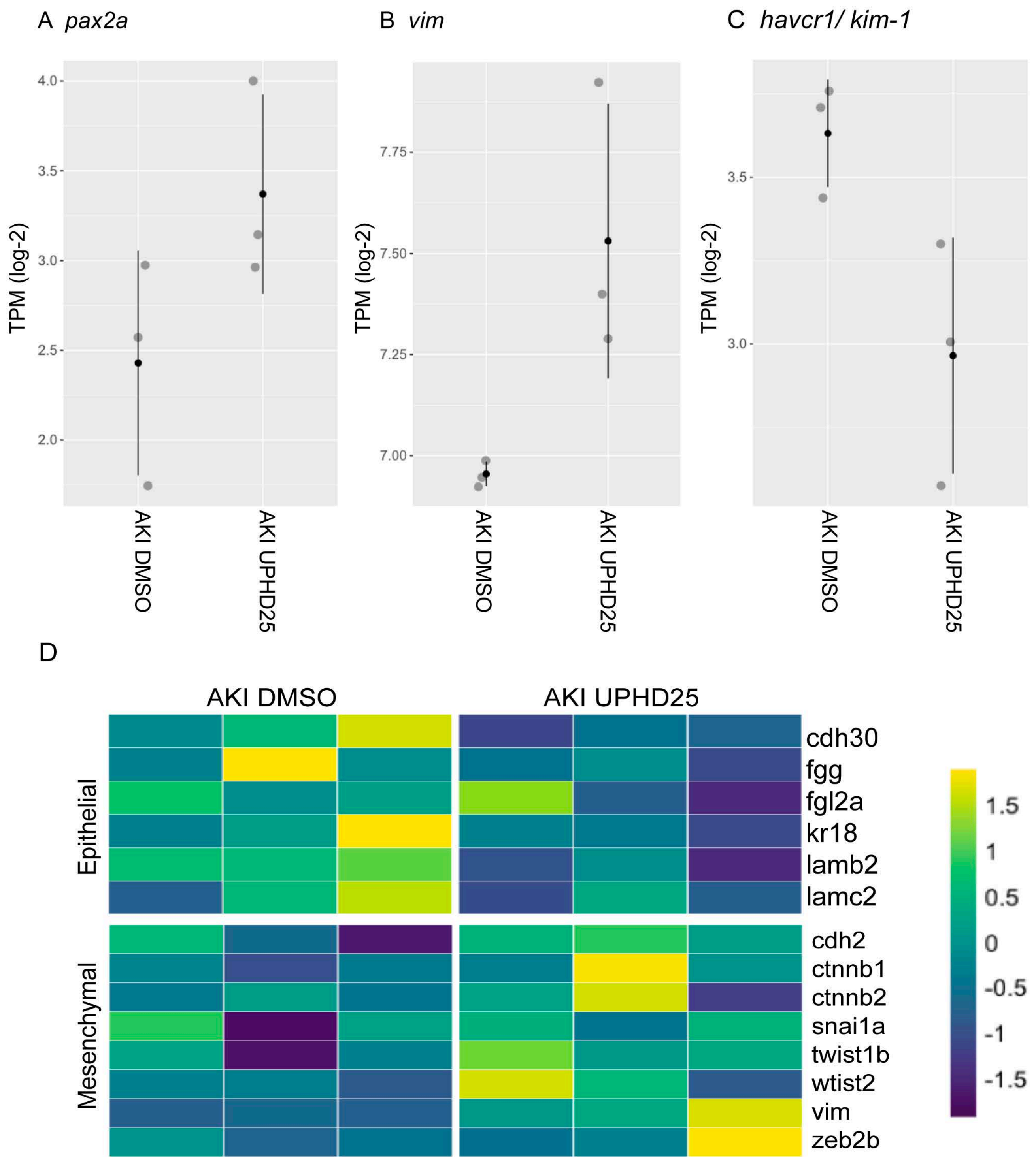
A pax2a
B vim
C havcr1/ kim-1
TPM (log-2)
4.0
3.5
3.0
2.5
2.0
7.75
7.50
7.25
7.00
AKI DMSO
AKI UPHD25
D
AKI DMSO
AKI UPHD25
Epithelial
Mesenchymal
cdh30
fgg
fgl2a
kr18
lamb2
lamc2
cdh2
ctnnb1
ctnnb2
snai1a
twist1b
wtist2
vim
zeb2b
1.5
1
0.5
0
-0.5
-1
-1.5

Figure S1. RNA-seq expression profiles in isolated injured tubules 6 dpf *Tg(PT:EGFP)* larvae were used to collect GFP+ pronephros of gent-AKI+DMSO, (AKI+DMSO) and gent-AKI +UPHD25 (AKI+UPHD25) at 3dpi. The tissues were used for RNA extraction then were sequenced All graphs were generated using transcripts -per-million, log2-scale. All treatments were replicated (N=3). (A) Transcript expression for *pax2a,* (B) Transcript expression for *vimentin,* (C) Transcript expression for *havcr1* (*kim-1*). All graphs were generated using transcripts -per-million, log2-scale. Each dot represents a biological replicate (grey) and the mean of total replicates (black). (D) Heatmap of RNA-seq expression levels of a panel of marker genes known to be critical in epithelial-to-mesenchymal (EMT) transition. Each column represents one biological replicate.

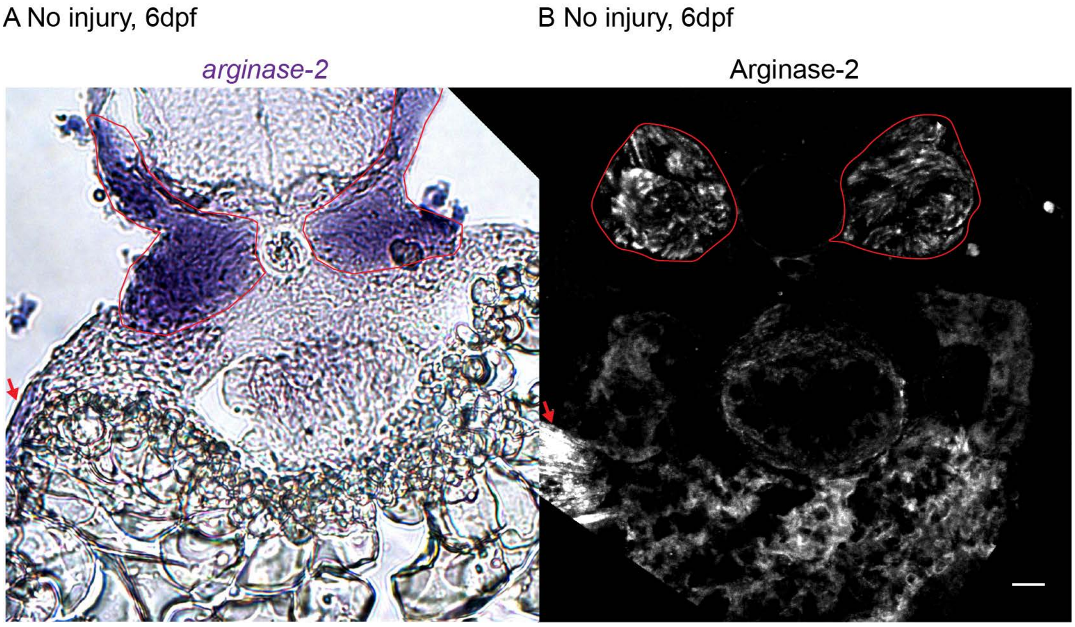

Figure S2. Validation of arginase-2 antibody via in situ hybridization

To validate the M2 marker arginase-2, we performed *in situ* hybridization to localize mRNA and compared the expression pattern to immunofluorescence staining with anti-arginase-2 antibody. (A) *In situ* hybridization of *arginase-2* on frozen section of 6 dpf uninjured larvae demonstrates positive staining in the somites (outlined in red) and pectoral fin (red arrow), consistent with previously reported spatiotemporal expression (zfin.org/ZDB-GENE-030131-1334). (B) Immunofluorescence staining in uninjured larvae at 6 dpf with anti-arginase-2 antibody (Thermo Fisher PA5-27987, 1:100) demonstrates congruent staining pattern, with positive staining in somites (outlined in red) and pectoral fin (red arrow). Scale bar= 20μm.

Disease Models & Mechanisms • Supplementary information

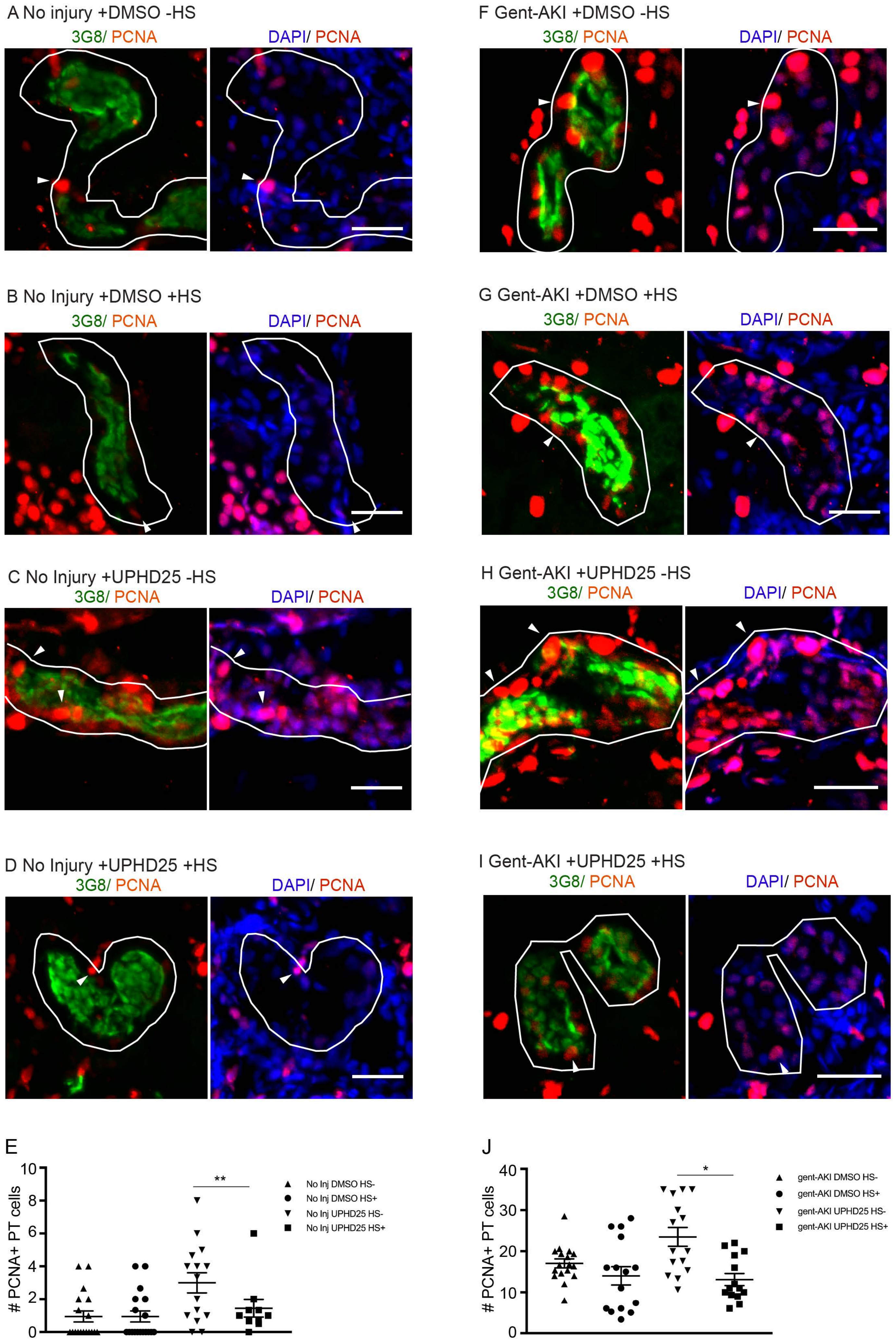
A No injury +DMSO -HS
3G8/ PCNA
DAPI/ PCNA
B No Injury +DMSO +HS
C No Injury +UPHD25 -HS
D No Injury +UPHD25 +HS
E
PCNA+ PT cells
**
No Inj DMSO HS-
No Inj DMSO HS+
No Inj UPHD25 HS-
No Inj UPHD25 HS+
F Gent-AKI +DMSO -HS
G Gent-AKI +DMSO +HS
H Gent-AKI +UPHD25 -HS
I Gent-AKI +UPHD25 +HS
J
*
gent-AKI DMSO HS-
gent-AKI DMSO HS+
gent-AKI UPHD25 HS-
gent-AKI UPHD25 HS+

Figure S3. Dominant negative RARα expression reduces UPHD25 efficacy Uninjured (A-E) or gent-AKI (F-J) *Tg(hsp70l:EGFP-HS-dnRARα)*^{ci1008} transgenic larvae were heat shocked at 5 dpf to express a dominant-negative form of human RA receptor alpha, which inhibits RA signaling. Larvae were treated with UPHD25 or DMSO then harvested for PT cell proliferation analysis (E, J). (A-H) Immunofluorescence for PCNA, marking proliferating cells in S-phase (red), 3G8, marking PT cells (green), and DAPI (blue) in uninjured fish treated with DMSO -HS (A) and +HS (B); uninjured fish treated with UPHD25 -HS(C) and +HS (D); gent-AKI fish treated with DMSO -HS (F) and +HS (G); and gent-AKI fish treated with UPHD25 -HS(H) and +HS (I). PT is outlined in white and arrows mark PCNA$^+$ RTECs. (E) Quantification of PCNA$^+$ RTECs in uninjured treatment groups. (J) Quantification of PCNA$^+$ RTECs in gent-AKI treatment groups. $\text{Mean}_{\text{Noinjury +UPHD25 -HS}}$=3.5 (N=15) vs. $\text{mean}_{\text{Noinjury +UPHD25 +HS}}$=1.5 (N=10) vs. $\text{mean}_{\text{gentAKI+UPHD25 -HS}}$=22 (N=15) vs. $\text{mean}_{\text{gentAKI +UPHD25 +HS}}$=12 (N= 14). Data pooled from 3 biological replicates are shown expressed as mean+/-SEM. One-way ANOVA: *$p<0.05$, **$p<0.01$. Scale bar=20 µm.

Disease Models & Mechanisms • Supplementary information

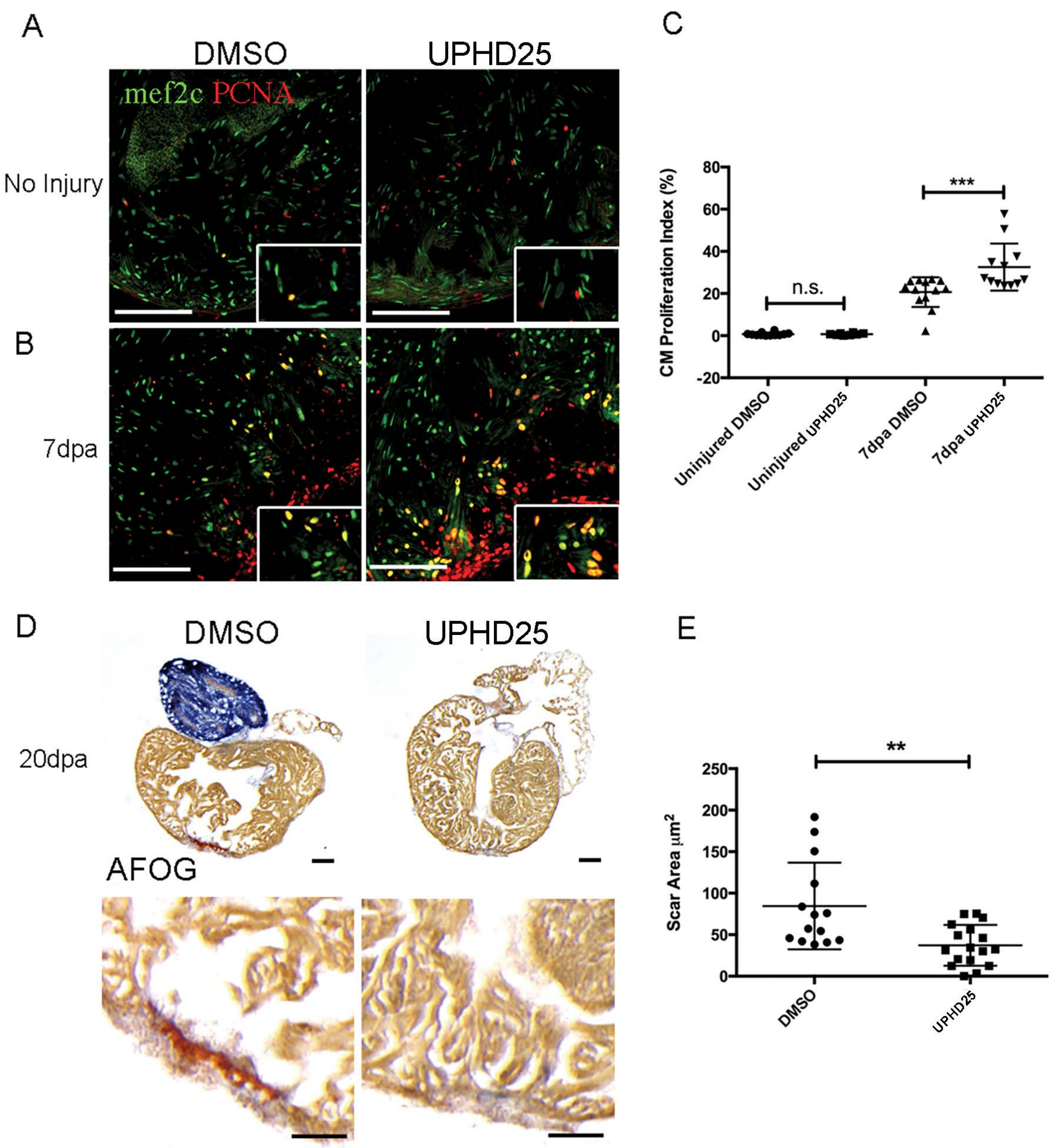

Figure S4. UPHD25 treatment ameliorates cardiac regeneration by promoting cardiomyocytes proliferation. (A) Mef2c and PCNA immunostaining of uninjured hearts, injected for 6 days with DMSO (N=12), or 0.4 mg/Kg UPHD25 (N=8). (B) Heart at 7 dpa injected with DMSO (N=9) and UPHD25 (N=8). (C) Quantification of cardiomyocyte proliferation index in uninjured and 7 dpa hearts injected with UPHD25. (D) AFOG staining of hearts at 20 dpa injected with DMSO (N=14), and UPHD25 (N=17). Intact cardiac muscle (yellow-orange), fibrin (red), and collagen (blue). (E) Quantification of scar size at 20 dpa after injection of UPHD25. $Mean_{DMSO}$=84.54 (N=14) vs. $Mean_{UPHD25}$=37.12 (N=17). Data pooled from 3 biological replicates are shown expressed as mean +/-SEM. 2-tailed t-test: ** P<0.01. n.s., not significant. Scale bars=100 µm.

Disease Models & Mechanisms • Supplementary information

Table S1

Primary and secondary antibodies used for immunofluorescence and associated experimental conditions are provided.

**Primary Antibodies**						
**Antigen**	**Source**	**Cat #**	**Species**	**Dilution**	**Tissue**	**Ant retriev**
3G8	EXRC	3G8.2C11	Mouse	1:200	Frozen	None
Pax2a	GeneTex	GTX128127	Rabbit	1:200	Frozen	Citrate
GFP	Aves Labs	GFP-1020	Chicken	1:300	Frozen	None/ Citrate
KIM-1	R&D Systems	MAB1817	Rat	80μg/mL	Frozen	None
Dendra2	Origene	TA180094	Mouse	1:200	Frozen	None
TNFα	AnaSpec	AS-55383	Rabbit	1:100	Frozen	Citrate
Arginase-2	Thermo Fisher	PA5-27987	Rabbit	1:100	Frozen	Citrate
PCNA	Sigma	P8825	Mouse	1:3000 (kidney); 1:1000 (heart)	Frozen	Citrate
E-cadherin	BD Biosciences	610182	Mouse	1:200	Frozen	Citrate
Vimentin	Millipore	MAB3400	Mouse	1:100	Frozen	Trypsin
Mef2c	Santa Cruz	SC-365861	Mouse	1:500	Frozen	None

**Secondary Antibodies**						
**Antigen**	**Source**	**Cat #**	**Species**	**Dilution**	**Tissue**	**Conjugate**
Goat anti-mouse	Abcam	ab97035	Goat	1:1000	Frozen	Cy3
Goat anti-rabbit	Thermo Fisher	A-11008	Goat	1:1000	Frozen	Alex488
Goat anti-mouse	Thermo Fisher	A-11001	Goat	1:1000	Frozen	Alexa488
Goat anti-rat	Thermo Fisher	A-11007	Goat	1:1000	Frozen	Alexa594
Donkey anti-rabbit	Thermo Fisher	A-10042	Donkey	1:1000	Frozen	Alexa568
Goat anti-chicken	Thermo Fisher	A-11039	Goat	1:1000	Frozen	Alexa555
Goat anti-rabbit	Thermo Fisher	65-6120	Goat	1:1000	Frozen	HRP
Goat anti-mouse	Thermo Fisher	A-11005	Goat	1:1000	Frozen	Alexa594

**Antigen Retrieval**			
**Method**	**Reagents**	**Cat #**	**Protocol**
Trypsin	Trypsin from porcine pancreas	Sigma-Aldrich, T7168	Dissolve 1 tablet/100mL ddH2O. Incubate slides at 37C for 30 minutes
Sodium Citrate buffer	Citric Acid	Sigma-Aldrich, 251275	10mM Sodium citrate, 0.05% Tween-20, pH=6.0. Incubate slides at 97C for 30 minutes. Cool for 30 minutes at room temp
	Tween-20	Sigma-Aldrich, P1379	

Disease Models & Mechanisms • Supplementary information

SUPPLEMENTAL MOVIES

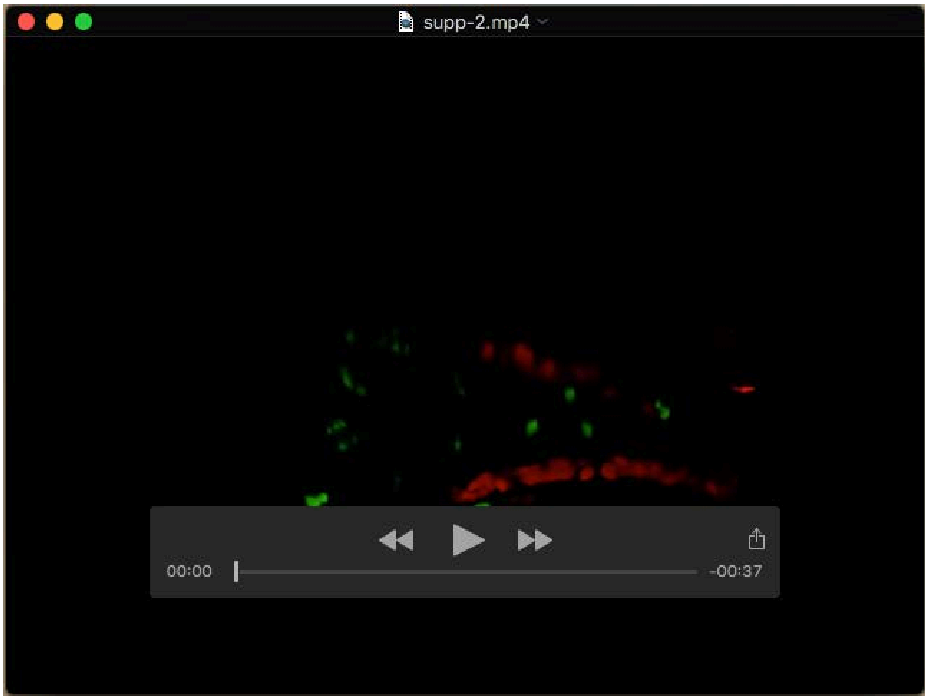

Movie 1

Uninjured *Tg(cdh17:mCherry);Tg(lyz:EGFP)* transgenic larvae were used to analyze neutrophil interaction (green) with PT (red) from 2.5-3.5 dpf. Neutrophils are observed circulating near the tubules, but without any prolonged contact with the PT. Leica TCS SP5 multiphoton microscope and HCXC IRAPO L 20X/0.95 water objective were used to capture images every 27 minutes for 17 hrs.

Disease Models & Mechanisms • Supplementary information

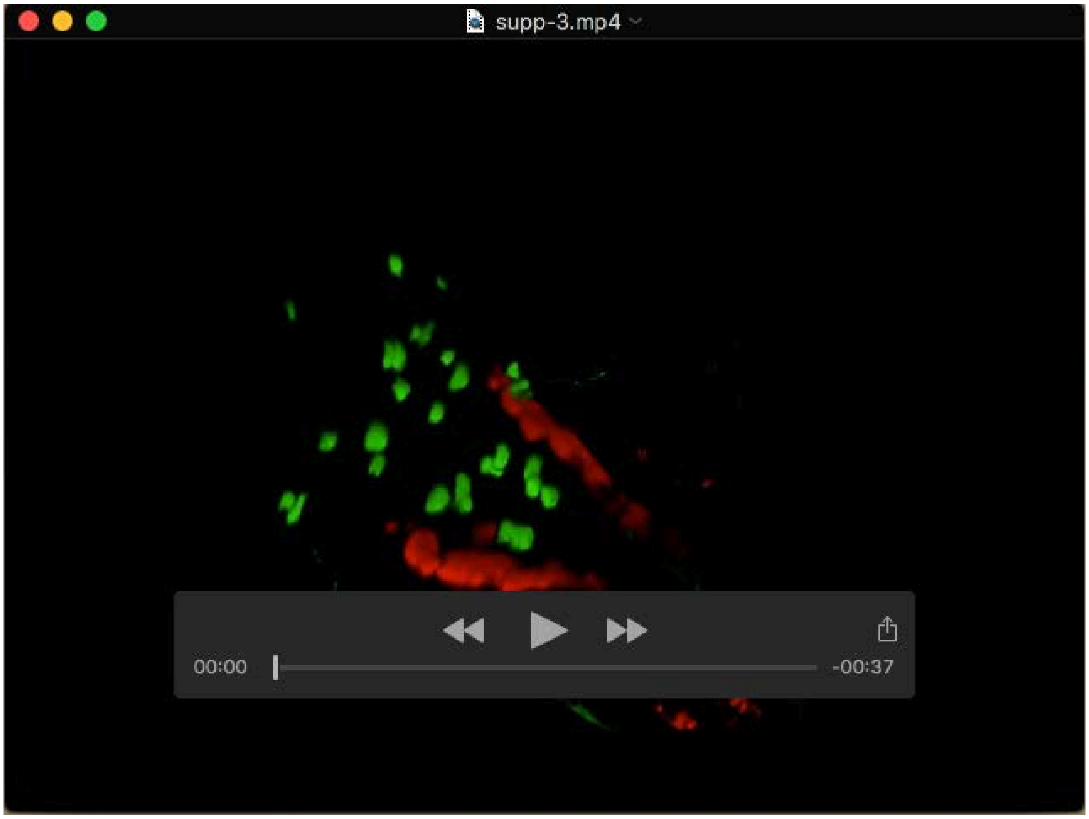

Movie 2

Gent-AKI *Tg(cdh17:mCherry);Tg(lyz:EGFP)* transgenic larvae were used to analyze neutrophil interaction (green) with PT (red) from 1-2 dpi. Higher number of neutrophils are observed near the tubules, with increased contact with the PT. Leica TCS SP5 multiphoton microscope and HCXC IRAPO L 20X/0.95 water objective were used to capture images every 27 minutes for 17 hrs.

Disease Models & Mechanisms • Supplementary information

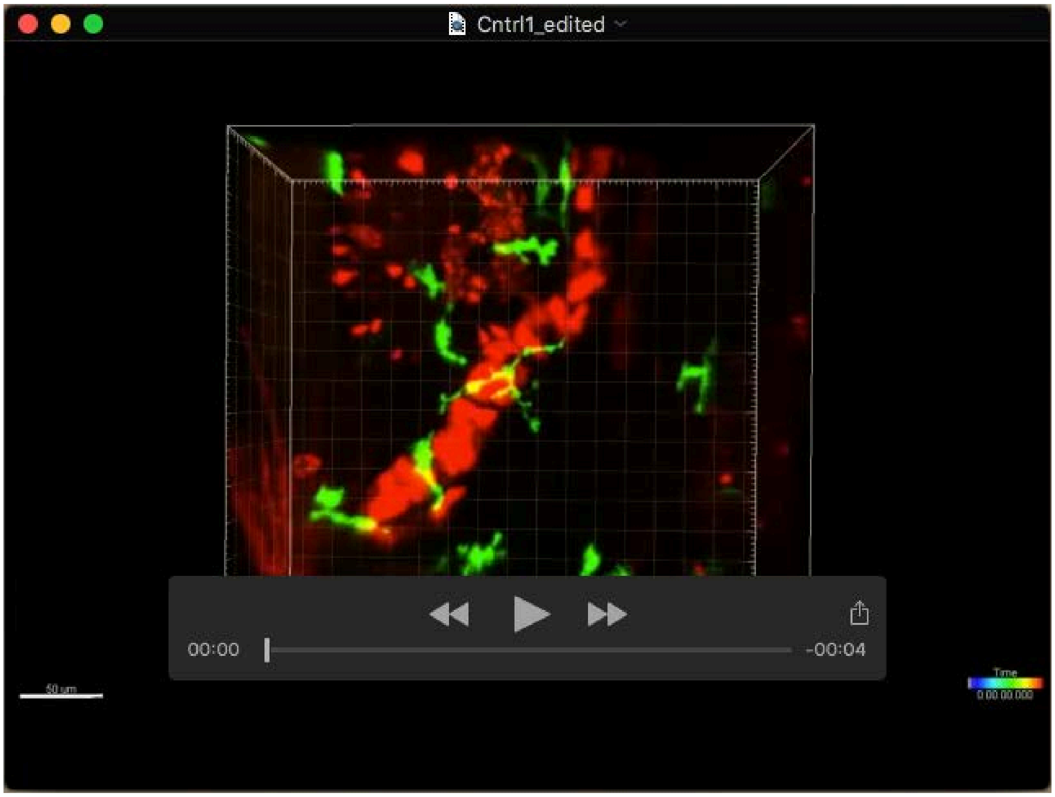

Movie 3

Uninjured *Tg(cdh17:mCherry);Tg(mpeg1:dendra2)* transgenic larvae were used to analyze macrophage interaction (green) with PT (red) from 4-5 dpf. Macrophages are observed circulating near the tubules, but without any prolonged contact with the PT. Nikon Eclipse Ti multiphoton microscope and 20X dry objective was used to capture images every 90 minutes for 24 hrs.

Disease Models & Mechanisms • Supplementary information

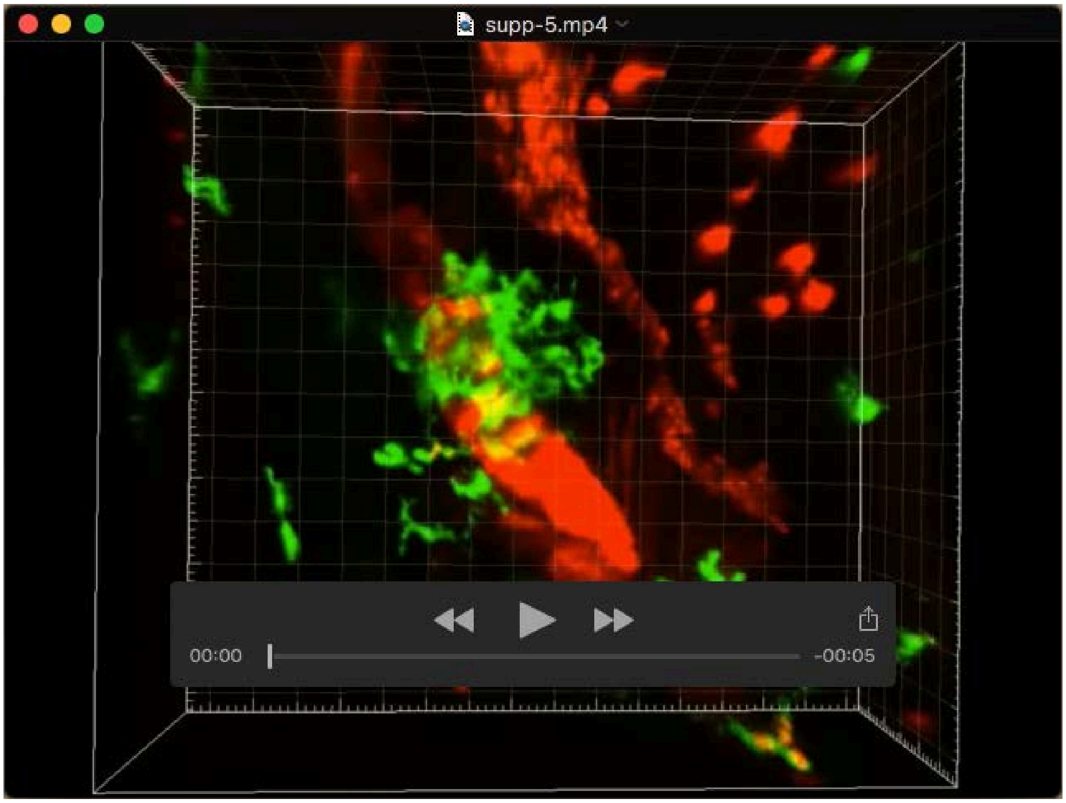

Movie 4

Gent-AKI *Tg(cdh17:mCherry);Tg(mpeg1:dendra2)* transgenic larvae were used to analyze macrophage interaction (green) with PT (red) in gent-AKI setting from 2-3 dpi. More macrophages are observed adjacent to the PT, with prolonged contact time. Nikon Eclipse Ti multiphoton microscope and 20X dry objective was used to capture images every 90 minutes for 24 hrs.

SUPPLEMENTAL METHODS

Zebrafish pronephros and RNA isolation

6 dpf *Tg(PT:EGFP)* larvae were used to collect GFP+ pronephros, 100 pronephros were used per condition, and all experiments were repeated three times. The larvae were incubated in 10mM DTT in E3 and Tricaine for 1.5-2 hrs at room temperature. The larvae were washed in Hank's Balanced Saline Solution (HBSS, Gibco). The larvae were incubated in 5mg/ml collagenase I in HBSS (Sigma Aldrich) for 2.5-3.5 hrs at 32 ºC. The larvae were washed in Minimum Essential Media and 10% fetal calf serum (MEM, FCS, Gibco). Using p100, the larvae were titurated to further digest the fish. Under a fluorescent scope, fine forceps and p10 pipette were used to dissect GPF+ pronephros to aspirate and collect pronephric kidneys from larvae on ice. The collected tubules were used for RNA isolation and sequencing. To collect total RNA, MEM+10% FCS was aspirated from pronephric tubule mass and then immediately processed with RNeasy Micro kit. (Qiagen). Tissue lysis and homogenization was performed in 500 µL Trizol (Thermofisher) and an electric homogenizer and pestle for 30 seconds. For phase separation, 50 µL BCP (Thermofisher) was added to the homogenate. Samples were centrifuged for 10 minutes at 12,000 rpm at 4C. 200 µL of top nucleic acid phase was collected and transferred into the column. Further downstream steps followed the manufacturer's standard protocol for the final elution of 10uL RNA. Total RNA quantification was performed using Nanodrop 1000 Spectrophotometer. Each condition was repeated with three biological replicates.

Library Preparation and RNA sequencing

1 µg of total RNA underwent mRNA library preparation using TruSeq Stranded mRNA kit (Illumina), according to the manufacturer's protocol. Fragmentation was performed for 8 minutes. 15 cycles of PCR amplification provided selective enrichment of DNA with adapters

Disease Models & Mechanisms • Supplementary information

ligated to both ends and was followed by library quantity and quality assessment by fluorometric assay (Qubit) and Agilent DNA 1000 TapeStation assay respectively. Final libraries were normalized to 10 nM, pooled and diluted. The sequence NextSeq 500 were seeded with 1.8 pM denatured library for automated cluster formation for approximately 30-40 million reads per sample. Four samples of the first biological replicates were pooled and sequenced on a single flow cell. Second and third replicates were pooled and ran on 2 flow cells.

***In situ* hybridization**

The *arginase-2* clone was synthesized and cloned into pEX-K248 with Sp6 promoter to drive the reverse transcription (Eurofins). The deoxygenin probe for *arginase-2* targeted 500 bp of 3' end of coding region and 500 bp of 3'UTR. 6 dpf larvae were fixed in 4% paraformaldehyde (PFA)/PBS overnight at 4°C. Larvae were washed in PBS. Larvae were dehydrated in of MeOH and then moved to -20°C for 1 hr. Larvae were transferred to acetone for 10 min at -20°C. Larvae were rehydrated in PBS and treated with 100 ug/mL Proteinase-K in PBS/0.2%BSA/0.1%Tween-20 (PBTw) for 30 min at RT. Larvae were again fixed in 4% PFA for 20 min. Larvae were incubated with the *arg-2* probe overnight at 65°C. Larvae were incubated in 1:2000 anti-DIG Alkaline Phosphatase antibody (Roche) overnight at 4°C. The larvae were stained with BM purple (Roche) at room temperature. The reaction was stopped with 4% PFA. Larvae were then cryosectioned (see protocol for immunofluorescence) and imaged using a 20X objective on an Axiovert 40 CFL brightfield scope (Zeiss). Images were captured using Axiovision Rel v4.8 software (Zeiss).

***Arginase-2* Antibody Staining**

Disease Models & Mechanisms • Supplementary information

6 dpf larvae were fixed with 4% PFA for 2 hours at room temperature. Larvae were washed with PBS and processed for sucrose gradient (10-20-30% PBS) then mounted with TFM (Ted Pella). The samples were cryosectioned at 14µm to obtain serial sections of PT. The sections were washed with PBSTw (0.1% Tween-20) and processed for antigen retrieval step using sodium citrate buffer for 15 minutes at 97.5°C. Slides were washed with PBSTw and blocked using 10% normal goat serum. They were incubated with rabbit anti-Arginase-2 (Thermo Fisher, PA5-27987) at 1:100 dilution, overnight at 4°C. The slides were washed and incubated with donkey anti-rabbit Alexa568 (Thermo Fisher, A-10042) at 1:1000 dilution, 2 hours at room temperature. The slides were washed and incubated with DAPI (1µg/mL) for nuclear staining (Millipore Sigma, D9542), 10 min at room temperature. The slides were washed with PBS then mounted with Aqua Polymount (Polysciences, 18606-20). Sections were imaged as described in the main methods.

Generation of transgenic line Tg(hsp70l:EGFP-HS-dnRARa)

Tg(hsp701:GFP-dn_Hsa.rarα) transgenic fish were generated by gateway-based Tol2 transposon transgenesis. To generate the transgenic fish, gateway cloning was used with the *hps70l* vector as the 5' entry clone and GFP-dnRARa, a human dominant negative RARa, as the middle entry. PolyA was used as the 3' entry clone. Following heat-shock induction, transgenic embryos expressed GFP and exhibited overt phenotypes, such as enlarged hearts and loss of forelimbs, which are consistent with loss of RA signaling. Constructs were injected into single cell embryos and screened for insertion. Progeny of P_0 founder animals were used to establish the zebrafish transgenic line. In order to examine the effect of dnRAR during AKI, 2 dpi or 5dpf zebrafish larvae were heat shocked at 37°C for 1 hour. Then, larvae were immediately treated with 1µM UPHD25 or 1% DMSO for 24 hours at 28°C. After treatment, the larvae were fixed and cryosectioned as

Disease Models & Mechanisms • Supplementary information

described in the main methods. The cryosections were then immunostained with rabbit anti-PCNA antibody as described in the main methods.

Cardiac injury and compound treatment

Adult AB* or Tu wildtype zebrafish age 6-18 months were anesthetized with 0.168 g/L tricaine for 3-5 minutes and ventricle apex amputation was performed as previously described (Missinato et al., 2018). In adults either 3 µL of 50% DMSO vehicle in PBS or 200 µM UPHD25 (corresponding to 0.4 mg/kg) was delivered by retro-orbital injection (Pugach et al., 2009). Injection was performed once per day, from 1-6 days post amputation (dpa), and hearts were extracted at 7 dpa to assess cardiomyocyte proliferation, and at 20 dpa to measure scar size.

Heart histologic analysis

Heart cryosections were stained with AFOG as previously described (Missinato et al., 2018). Images were captured with Leica MZ 16 microscope and Q Imaging Retige 1300 camera. Clot area was measured using ImageJ (NIH). Four heart sections showing the largest clot were measured and the scar area values were measured as average of the sum of the clot area. Slides were mounted in Vectashield with DAPI (Vector Laboratories). For each experiment, at least four sections were analyzed per heart. Cardiomyocyte proliferation index was calculated as percentage of number of $Mef2c^{+}$ and $PCNA^{+}$ cells divided by the number of total $Mef2c^{+}$ cells.

Missinato, M. A., Saydmohammed, M., Zuppo, D. A., Rao, K. S., Opie, G. W., Kuhn, B. and Tsang, M. (2018). Dusp6 attenuates Ras/MAPK signaling to limit zebrafish heart regeneration. *Development* **145**.

Pugach, E. K., Li, P., White, R. and Zon, L. (2009). Retro-orbital injection in adult zebrafish. *J Vis Exp*.
